# Towards *In Silico* Identification of Genes Contributing to Similarity of Patients’ Multi-Omics Profiles: A Case Study of Acute Myeloid Leukemia

**DOI:** 10.3390/genes14091795

**Published:** 2023-09-13

**Authors:** Declan J. Batten, Jonathan J. Crofts, Nadia Chuzhanova

**Affiliations:** School of Science and Technology, Nottingham Trent University, Clifton Lane, Nottingham NG11 8NS, UK; declan.batten2016@my.ntu.ac.uk (D.J.B.); jonathan.crofts@ntu.ac.uk (J.J.C.)

**Keywords:** similarity network fusion (SNF), spectral clustering, chromosome conformation (Hi-C) data, multi-omics data, mutations, protein–protein interactions (PPI), acute myeloid leukemia (AML)

## Abstract

We propose a computational framework for selecting biologically plausible genes identified by clustering of multi-omics data that reveal patients’ similarity, thus giving researchers a more comprehensive view on any given disease. We employ spectral clustering of a similarity network created by fusion of three similarity networks, based on mRNA expression of immune genes, miRNA expression and DNA methylation data, using SNF_v2.1 software. For each cluster, we rank multi-omics features, ensuring the best separation between clusters, and select the top-ranked features that preserve clustering. To find genes targeted by DNA methylation and miRNAs found in the top-ranked features, we use chromosome-conformation capture data and miRNet2.0 software, respectively. To identify informative genes, these combined sets of target genes are analyzed in terms of their enrichment in somatic/germline mutations, GO biological processes/pathways terms and known sets of genes considered to be important in relation to a given disease, as recorded in the Molecular Signature Database from GSEA. The protein–protein interaction (PPI) networks were analyzed to identify genes that are hubs of PPI networks. We used data recorded in The Cancer Genome Atlas for patients with acute myeloid leukemia to demonstrate our approach, and discuss our findings in the context of results in the literature.

## 1. Introduction

The advent of new technologies makes multi-omics (mRNA and micro-RNA expression, DNA methylation) and mutational profiles routinely available for each individual patient. These data could potentially give clinicians and researchers a more comprehensive view on any given disease and allow them to more accurately assess existing similarities/differences between patients in terms of their omics, mutational profiles and other (clinical) features. Identification of patients’ similarities/differences, especially for highly heterogeneous diseases, finding informative features that contribute to these similarities and, most importantly, suggesting plausible biological interpretation for observed similarities is invaluable for a better understanding of the molecular basis of a given disease and patients’ survival trajectories. It may also lead to the discovery of novel therapeutic and diagnostic targets for optimized treatment and disease management for specific groups of patients.

In this paper, we focus on finding informative and biologically plausible features contributing to the similarities of patients, as identified by clustering of multi-omics profiles of patients with Acute Myeloid Leukemia (AML), known to be a highly heterogeneous disease from both a biological and clinical point of view [1]. 

AML is the most common type of acute leukemia affecting adults. It accounts for 2500 annual fatalities in the United Kingdom (Cancer Research UK, 2017), which is almost seven deaths daily. Currently AML is cured in only 35–40% and 5–15% of patients under and over 60 years of age, respectively. Although morphological and immunophenotypic, cytogenetic and other molecular alterations incorporated in the FAB (French-American-British) and ELN (European Leukemia Net) genetic risk stratification systems allow us to predict individual clinical trajectories, their accuracy remains relatively low, since between 50% and 70% of AML patients harbor normal or risk-indeterminate karyotypes [2]. The most recent edition of the World Health Organization (WHO) classification of AML, which is based on therapeutically and/or prognostically assessable biomarkers [3], may lead to a more accurate stratification of patients. 

Various *in silico* (clustering) procedures have been used for guiding the identification of prognostic signatures, based on either expression of a specific set of genes/immune genes with or without clinical features and/or multi-omics data such as gene and miRNA expression and DNA methylation. Among them is a prognostic signature based on the expression of 66 genes proposed by Metzeler et al. [4]. A score based on this signature shows a correlation with overall survival (OS; i.e., dead or alive) in cytogenetically normal AML patients. A 17-gene leukemia stem cell score (LSC17) proposed by Ng et al. [5] proved to be accurate in predicting resistance to chemotherapy. High LSC17 scores were found to be good predictors of poor outcome of current treatments including allogeneic stem cell transplantation, thus identifying a cohort of AML patients who will not benefit from standard therapies. Wagner et al. [2] proposed a 3-gene signature based on expression of the *CALCRL*, *CD109* and *LSP1* genes. This 3-gene-based prognostic index allows the identification of a finer classification within each ELN cytogenetic risk category into subgroups with different survival probabilities. This signature, together with other features (e.g., FLT3 internal tandem duplication or non-mutated nucleophosmin 1, *NPM1*), was predictive of event-free and OS of AML patients. The prognostic role of immune genes in AML was explored by Zhu et al. [6]. In total, 136 immune-related genes associated with OS in AML patients were identified. In turn, the protein–protein interaction (PPI) analysis of these genes resulted in identifying a subset of 24 immune-related genes as being hubs of the PPI network. This subset of genes was further reduced to six immune genes: *CSK*, *MMP7*, *PSMA7*, *PDCD1*, *IKBKG* and *ISG15*. These genes were subsequently used to create an immune-related gene signature for stratifying AML patients into two groups corresponding to OS. Figueroa et al. [7] used DNA methylation profiles of 344 patients with AML to identify 16 groups. Interestingly, five of the groups found were new AML subtypes and did not share any known features. Subsequently, a 15-gene methylation signature was created allowing the prediction of OS in an independent patient cohort (*p* < 0.001, adjusted for known covariates). A multi-omics profiling framework based on the integration of similarity network fusion [8] and machine learning techniques was proposed by Chierici at al. [9]. For 157 AML patients, several features such as gene expression, miRNA expression and DNA methylation available from TCGA repository were used to predict the OS. Although the proposed approach showed the best precision/accuracy balance in comparison with other approaches in the 10 × 5-fold cross validation settings (Matthew correlation coefficient, MCC = 0.274), this value was much lower than the corresponding values for, e.g., the BRCA subtyping (MCC = 0.788) and BRCA estrogen receptor status (MCC = 0.820), prompting the need for novel and more accurate approaches for identifying features predictive of AML patients’ overall survival, incorporating multi-omics and other available data.

In this paper, we propose a computational framework that reveals informative and biologically plausible genes among features identified by any relevant clustering procedure as contributing to patients’ similarity. This framework is based on integrating multi-omics data with chromosome-conformation capture (Hi-C) data, somatic/germline mutations and knowledge of PPI. We use data recorded in The Cancer Genome Atlas (TCGA; https://www.cancer.gov/ccg/research/genome-sequencing/tcga, accessed on 20 November 2019) database for patients with AML. First, the similarity network fusion (SNF, [8]) was used to identify patients’ fused similarity network based on three types of multi-omics data: mRNA expression of immune genes, miRNA expression and DNA methylation. Signatures based on immune genes have been validated before and proved to be important for survival prediction in several types of cancers (see, for example, [10]). Second, we used spectral clustering to identify the optimal partition of the resulting fused network into homogeneous patients’ subgroups/clusters. To identify informative features contributing to similarity of patients’ multi-omics profiles, for each cluster we ranked multi-omics features, ensuring the best separation of a given cluster from the remaining ones. For each cluster, we selected top-ranked features that, when used for creating fused similarity network and spectral clustering, ensure 95% accuracy, i.e., classify 95% of patients into the same clusters as in the original clustering based on all features. Immune genes found in the selected top-ranked features in each cluster were combined with immune genes that may be targeted by methylated DNA loci and miRNAs found among those selected top-ranked features. For the former, we used chromosome-conformation capture (Hi-C) data [11]; for the latter, the miRNet2.0 [12] software tools were used. To identify subsets of immune genes contributing to the similarity/homogeneity of patients within each cluster and investigate the plausibility of these genes playing a role in AML, these combined sets of immune genes were explored in terms of their enrichment in somatic/germline mutations and GO biological processes/pathways terms. The Molecular Signature Database from GSEA was used to assess the enrichment of these combined sets in several known gene sets often considered to be important in relation to AML. The PPI analysis of the sets of immune genes was performed with the aim of identifying genes that are hubs of PPI networks and, when dysregulated, may influence the largest number of interacting proteins. We discussed our findings in the context of results published so far, although detailed biological interpretation of the identified genes was beyond the scope of this paper.

## 2. Materials and Methods

### 2.1. Publicly Available Datasets Used in This Study

The list of immune genes was compiled from the following databases: the Immunology Database and Analysis Portal (ImmPort; 4723 genes; https://www.immport.org/, accessed on 20 November 2019), the Innate Immunity Genes database (InnateDB; 1378 genes; http://www.innatedb.com/, accessed on 20 November 2019), data from the Davis et al. (2005) paper [13] (3148 genes), the Immunogenetic-Related Information Source (IRIS) [14] containing 1508 immune genes and the Immunome database [15] (824 genes; http://structure.bmc.lu.se/idbase/Immunome/, accessed on 20 November 2019). After removing duplicates, 7810 immune genes and gene transcripts were retained. 

The NCBI Reference Sequence Database (RefSeq; https://www.ncbi.nlm.nih.gov/refseq/, accessed on 20 November 2019) was used to identify genes and their positions in the GRCh37/hg19 assembly. In total, there were 51,537 gene transcripts of 26,368 genes, henceforth referred to as RefSeq genes. 

We used single nucleotide polymorphisms (SNPs) identified by the GWA study of 300 AML samples [16]. For each SNP, its linkage disequilibrium (LD) block, available in Lv et al. (2017) [17], was used. The dataset of 1754 LD blocks was downloaded from the GWAS Catalog (https://www.ebi.ac.uk/gwas/studies/GCST008413, accessed on 20 November 2019). 

The dataset of somatic mutations in 200 AML patients was downloaded from TCGA database (https://www.cancer.gov/ccg/research/genome-sequencing/tcga, accessed on 20 November 2019). It contained a total of 2749 mutations in 2275 RefSeq genes and genomic transcripts including 1072 mutations in 871 immune genes.

Chromosome conformation capture (Hi-C) data for all-trans retinoic acid (ATRA)-induced HL-60 cells, frequently used as a model of leukemia cell differentiation as reported in Li et al. (2018) [11], were downloaded from the GEO database (four datasets; accession number GSE93997). These datasets contained ~175 million interactions which we have subsequently binned into 40 K regions and counted the frequency of interactions between these 40 K regions. To identify the strongest intra-chromosomal interactions, distributions of interacting frequencies were analyzed individually for each chromosome. For each chromosome, only interactions (and corresponding 40 K bins) recorded in all four datasets and residing within the tails of these distributions that correspond to 5% of all interactions were considered to be the strongest intra-chromosomal interactions. For inter-chromosomal interactions, only interactions and their corresponding bins found in all four datasets were deemed to be significant and used in further analysis. Note that the frequency of intra- and inter-chromosomal interactions between two loci is inversely proportional to their closeness within the cell nucleus.

DNA methylation, miRNA expression and mRNA gene expression data were downloaded from TCGA database (https://portal.gdc.cancer.gov/repository, accessed on 20 November 2019) for a cohort of 200 AML patients. Methylation levels at 27,578 CpG loci across the genome were recorded as conventional β values. The mRNA expression of 51,537 genes and gene transcripts was measured in terms of FPKM (fragments per kilobase of transcript per million mapped reads) values that consider the length of the gene sequence to account for biases in the sequencing process. For miRNA expression, RPM (reads per million mapped reads) values that take into account the number of reads mapped to miRNAs and the total number of miRNAs (which was 1881), were downloaded. Patients that exhibited greater than 20% of data missing in a single omics data type were removed. Further, features (either methylation loci, miRNAs or genes/gene transcripts) were omitted if they exhibited greater than 20% missing data across all patients and data types. The remaining missing values were imputed using a K-nearest-neighbor approach (K = 20) based on weighted averages; all data were further normalized using log2 and Z-transform. The resulting datasets for 112 patients consisted of methylation levels at 24,889 loci, expression of 415 miRNAs and 51,537 genes/gene transcripts.

Positions of LD blocks, genes and mutations were binned into corresponding 40 K region(s). When required, genomic positions of features were ‘lifted over’ to the GRCh37/hg19 assembly, using the Lift Genome Annotation program available at https://genome.ucsc.edu/cgi-bin/hgLiftOver (accessed on 20 November 2019). 

Clinical data for each of 112 patients were downloaded from TCGA database and is summarized in Table S1. Note that all datasets were downloaded between October and December 2019 when this study commenced.

### 2.2. Similarity Network Fusion (SNF)

Similarity Network Fusion (SNF) implements a sophisticated method of integrating multi-omics data based on message-passing theory [8]. SNF initially constructs similarity matrices for each available data type (e.g., mRNA and miRNA expression, DNA methylation) using the Euclidean distance function and a scaled exponential similarity kernel. Then, SNF uses a K-nearest-neighbor approach to form sparse local similarity matrices that encode the local community structure of the patient similarity network corresponding to each similarity matrix. It assumes that local similarities are more reliable information sources than similarities between patients that do not share the same local neighborhood, since those similarities form weak connections in the patient similarity network. The integration stage involves iteratively updating the full similarity matrices using the local similarity information encoded in the sparse kernel matrices. This is performed using a non-linear combination method that has its roots in message-passing and label propagation theory [18]. The final similarity matrices were shown to converge [8] after a suitable number of iterations (experiments have shown that 20 iterations suffice in most cases). The data integration stage is finalized by forming a similarity matrix whose entries are defined as the average of the corresponding entries in each of the final similarity matrices.

A comparison of several multi-omics integration approaches performed by Tini [19] demonstrated that SNF is the most robust method for analyzing complex datasets. It exhibits a unique ability to detect complementary signals and is robust to noise (as it uses reliable local similarity information throughout the integration stage). Furthermore, since the local information (which represents strong similarity connections) is propagated to all similarity matrices in the integration stage, strong similarities are emphasized in the final fused matrix. Hence, SNF exhibits the unique property that it up-weights strong connections and down-weights weak connections in the patient similarity network, which often results in more clearly defined and well-separated patient communities that can easily be captured using spectral graph partitioning techniques.

Following data integration, SNF employs spectral clustering using the NCut algorithm [20] to determine the optimal partition of the network into homogeneous patient subgroups. Spectral clustering partitions the similarity network by using graph-theoretical properties of the similarity network. In turn, NCut aims to minimize between-cluster similarity whilst maximizing the similarity within the same cluster (for more detailed definitions see, e.g., [8]). To compute the optimal number of clusters, we used the EigenGap approach and RotationCost, adopted by SNF.

### 2.3. Identifying Informative Features Contributing to Cluster Separation

Following Wang et al. [8], we ranked features according to their significance in each cluster using a ranking similar to the Normalized Differential Expression (NDE) index suggested by Tusher [21]. A feature in the *k*th cluster was considered to be significant if its average value in that cluster was different from the average value calculated across all other clusters:

$$NDE=\frac{\left|\mu \left(Cl_{k}\right)-\mu \left({\bar{Cl}}_{k}\right)\right|}{\sqrt{|Var\left(Cl_{k}\right)-Var\left({\bar{Cl}}_{k}\right)}}\;\frac{\frac{1}{m_{k}}+\frac{1}{n-m_{k}}}{n-2}$$

Here $\mu \left(Cl_{k}\right)$ and $\mu \left({\bar{Cl}}_{k}\right)$ ($Var\left(Cl_{k}\right)$ and $Var\left({\bar{Cl}}_{k}\right)$) are average values (variances) of a given feature in cluster *k* and the rest of the patients, respectively; $m_{k}\;$ and *n* are the number of patients in cluster *k* and the entire dataset, respectively.

In total, 33,114 features (415 miRNA expression, 7810 immune gene expressions and DNA methylation levels of 24,889 loci) available for 112 patients were considered for ranking. Finally, we constructed a list of features in descending order of their corresponding NDE values. Features at the top of this list were considered to be more important/informative for separating patients in the *k*th cluster from the rest of the patients.

### 2.4. Other Software Packages Used

The software package miRNet2.0—a miRNA-centric network visual analytics platform—described in Chang [12] and available at https://www.mirnet.ca/miRNet/home.xhtml (accessed on 20 November 2019), was used to identify potential target genes of miRNAs. Enrichment analyses were performed using Metascape (https://metascape.org/gp/index.html, accessed on 20 November 2019). A few selected gene sets corresponding to the hallmark interferon IFN-α (M5911) and IFN-γ (M5913) response and inflammatory response (M5932) were downloaded from the Molecular Signature Database [22,23], available via the GSEA website (https://www.gsea-msigdb.org/gsea/msigdb/, accessed on 20 November 2019). Protein–protein interaction data from the STRING database (https://string-db.org/, accessed on 20 November 2019) were used to create PPI networks for selected gene sets. Node degree distributions were used to identify the top 5% (1%) genes with the highest number of interactions with other proteins; these genes were considered to be hubs of the networks.

## 3. Results

### 3.1. SNF Analysis

First, patient similarity matrices (networks) for each multi-omics dataset—DNA methylation, mRNA expression of immune genes and miRNA expression—were fused together using SNF_v2.1 software and then clustered using spectral clustering by the NCut algorithm [8]. The optimal number of clusters for each data type was assessed by the RotationCost and EigenGap approaches and varied between two and four. Supplementary Figure S1 shows the results for patient clustering using single omics datasets.

It is apparent that these clusters (with exception of a few) exhibit relatively low within-cluster similarity. For mRNA gene expression, no visible patient clusters were identified (Supplementary Figure S1a,b). For DNA methylation and miRNA expression, one and two patient clusters, respectively, with higher within-cluster similarity were emerging (Supplementary Figure S1c,d). The between-cluster similarity was generally lower than within-cluster similarity, suggesting some degree of separation between those clusters with respect to a single omics dataset. 

**Figure 1:**
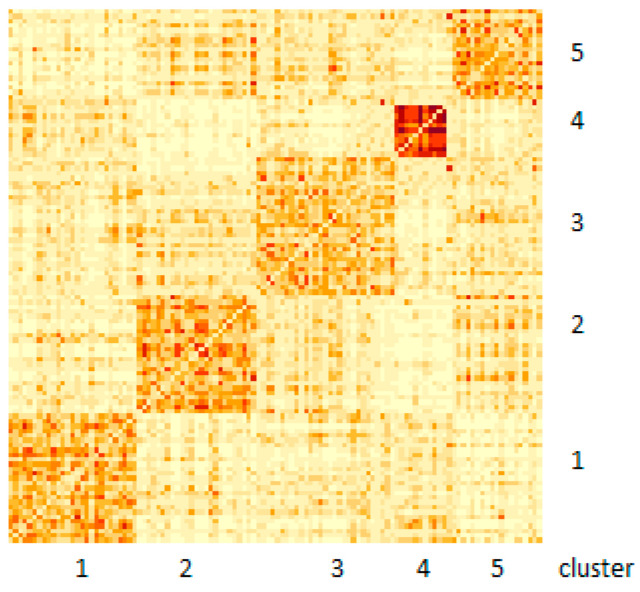
Heatmap of fused similarity network following spectral clustering into five clusters. Darker colors indicate higher similarity.

Further, patient similarity networks for each data type were fused together using the SNF_v2.1 software [8], with default parameter settings (α = 0.5, T = 20 and K = 20). The fused similarity network was subjected to spectral clustering by NCut. The number of clusters was set to five. This number of clusters was found as an optimal number among first- and second-best choices by the RotationCost and EigenGap approaches, respectively. The heatmap in Figure 1 displays the results of the patient clustering (see also Table 1 for cluster sizes and their characteristics). The heatmap shows a generally good cluster separation and exhibits lower between-cluster similarity as compared to the clustering obtained using single omics datasets. 

Cluster 4 is the smallest (11 patients) and most homogeneous cluster; it has the strongest within-cluster similarity in the network. Further, all patients in this cluster characterized as the FAB M3 AML subtype, i.e., acute promyelocytic leukemia (see Table 1). According to the ELN risk categories, every patient in Cluster 4 (with one exception) exhibits a favorable prognosis. Further, only 11 patients in the entire dataset were characterized as the FAB M5 subtype (acute monocytic leukemia); all these patients were found in Cluster 2 (Table 1). Twenty out of 25 patients in this cluster were classified as having intermediate/normal prognosis according to the ELN risk categories. Mutations in the *NPM1c* gene were found in almost half of patients in Clusters 2 (14/25) and 5 (9/20) (see Supplementary Table S2). Interestingly, the FAB M3 subtype was detected in the absence of any cytogenetic/chromosomal lesion information during data integration and clustering, which suggests the involvement of other biological processes/features in this disease subtype. Other clusters, however, did not significantly associate with either a particular category of existing classification systems or exhibit any distinct cytogenetic or immunophenotypic homogeneity (see Supplementary Table S2), suggesting the involvement of other biological modalities in defining patients’ similarity. However, it was noted that patients from poor-risk and favorable-risk categories were rarely clustered together (Table 1). To explore it further, the survival analysis was performed. 

### 3.2. Survival Analysis

**Figure 2:**
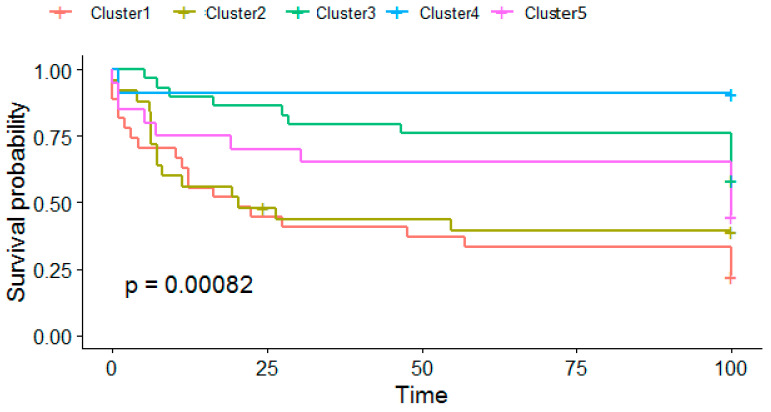
The Kaplan–Meier survival curves for each of five patient clusters identified.

To find out whether the survival curves are different for patient clusters identified using spectral clustering of the fused network, the Cox log-rank test was performed. The test indicated that there is evidence to suggest that there is a significant (*p* = 0.00082) difference between the survival profiles of patients in the clusters found (Figure 2). The Kaplan–Meier curve for Cluster 4 showed the highest survival probability. This aligns well with the ELN risk classification of patients in this cluster; all patients in Cluster 4 (with one exception) exhibit a favorable prognosis. Approximately 83% of patients in Cluster 3 were classified as having intermediate/normal and favorable prognosis according to the ELN risk classification, whereas all patients in Cluster 1 are classified as having poor or intermediate/normal prognosis. In general, there is a good agreement between the rankings of survival probabilities and the proportion of patients in each cluster having favorable prognosis (Spearman correlation = 0.82), although the *p*-value = 0.08859 is slightly higher than the 5% level of significance.

### 3.3. Informative Feature Selection

To find features that make the largest contribution to the patients’ survival/risk category prediction, as captured by clustering of the fused similarity network, a ranked list of all 33,114 features comprising 415 miRNA, 7810 immune genes/transcripts and 24,889 DNA methylation loci was constructed for each cluster using the NDE index (see Section 2). The values of the NDE index, which, by definition, indicate the potential of each feature to separate patients of a given cluster from the rest of the patients, decrease dramatically even within the top 100–200 ranked features. For many features, especially at the bottom of these rankings, the NDE values are identical, showing that these features make similar, albeit small, contributions to cluster separation. We found that using the top 125 features from each cluster (613 unique features in total across all clusters) allowed us to produce clustering similar (with 95% accuracy) to the one obtained using all 33,114 features. Therefore, we speculate that these top-ranked 125 features from each cluster make the most contribution to cluster separation and could be considered as the most important and informative ones.

The majority (92–98%) of features appearing among the top 125 in each cluster were DNA methylation loci. To find immune genes that could be potential targets of the observed DNA methylation (i.e., their expression may be affected by DNA methylation either via intra- or inter-chromosomal interaction between methylated loci and gene promoter/gene), intra- and inter-chromosomal Hi-C interactions data (see Section 2 for details) were used. For each cluster, we compiled a list of immune genes that could be potential targets of methylation. Further, immune genes listed in TCGA database as potential targets of methylation loci, typically residing within close proximity to the methylation sites but not found following the stringent definition of “strong” intra-chromosomal interaction (see Section 2), were added to the corresponding lists. 

Between six and 15 miRNAs were also found among top-ranked features. Immune genes potentially targeted by these miRNAs were found using miRNet [12] and added to the compiled lists of genes. Immune genes found in the top 125 most informative features were also kept. 

Using the selection procedures described above, the sets of 1542, 1927, 1328, 1720 and 2782 immune genes (SIGs for short) that may play a role in AML were identified in Clusters 1 to 5, respectively (see Supplementary Table S3). The overall number of different immune genes (including target genes) across all five clusters was 4692, i.e., 60% of all immune genes (7810) used in this study. Note that it is unlikely that all the genes identified using inter- and intra-chromosomal interactions between 40 K fragments play a role in AML; some of them may be simply “bystanders”, which happened to occur within the same 40 K regions harboring genes that may play a role in the development of AML. Although a systematic biological interpretation of genes in the identified SIGs was beyond the scope of this paper, we explored their enrichment in GO terms and genes from curated datasets, presence of somatic mutations, occurrence of genes prone to germline mutations (as identified by the GWA study of unrelated AML patients) and presence of genes being hubs of PPI network, with the aim of identifying biologically plausible features contributing to patients’ similarity.

### 3.4. Enrichment Analysis

For each of five SIGs identified by selection procedures described above, their enrichment in GO biological processes and pathways terms was performed using Metascape. The SIGs were found to be enriched in ‘cytokine signaling in immune system’ reactome GO term; *p*-values corrected for multiple testing (i.e., q-values) were between ~10^−96^ and 10^−41^ in all five clusters. The SIGs were also enriched in ‘signaling by interleukin’ reactome term (10^−76^ < q < 10^−31^), ‘apoptotic signaling pathway’ GO term (10^−76^ < q < 10^−40^) and many others; for the full lists, see Supplementary Table S4. 

Using the Molecular Signature Database from GSEA, we downloaded curated sets M5911, M5913 and M5932 containing genes that are up-regulated in response to α and γ interferon proteins and defining inflammatory response in AML, respectively. 

The numbers of immune genes shared between SIGs for each cluster and gene lists corresponding to GO terms or curated datasets analyzed are summarized in Table 2. Some of the immune genes were present in several SIGs (i.e., the total number of unique genes is not a sum of genes in all clusters), but the majority of them were unique to each cluster. 

### 3.5. Germline and Somatic Mutations in Immune Genes

A dataset of somatic mutations together with their potential target genes was downloaded from TCGA database and consisted of 2749 mutations occurring or residing within close proximity to 2275 RefSeq genes. The most frequently mutated gene was *DNMT3A*, with 33 mutations in 30 out of 200 AML patients. The next most frequently mutated genes were immune genes *TP53* and *FLT3*; 11 and 13 patients had mutations in these genes, respectively. The majority of mutated genes were harboring between one and four somatic mutations per patient. Out of 7810 immune genes used in this study, 871 genes were harboring 1072 mutations. In a subset of 112 patients selected for this study, 680 somatic mutations were recorded in 581 immune genes.

The dataset of 1754 LD blocks [17] encompassing germline mutations (SNPs) identified by GWA studies [16] in patients unrelated to TCGA AML cohort were found to reside within 1124 distinct 40 K bins. In total, 712 RefSeq genes including 242 immune genes were either harboring or residing within LD blocks corresponding to at least one SNP showing genome-wide significance (*p* < 5 × 10^−6^). 

We found that immune genes were enriched in both germline and somatic mutations (Fisher’s Exact Test, *p* < 2.2 × 10^−16^) as compared to non-immune genes. 

It is known that chromatin interactions could bring together genes and germline mutations that are not necessarily within the same LD block. We used Hi-C data to identify possible intra- and inter-chromosomal regions strongly (as described in Section 2) interacting with regions harboring germline mutations. For intra-chromosomal interactions, the cut-off interaction frequency corresponding to approximately 5% of the strongest interactions across various chromosomes was found to be 100. Genes residing within these interacting regions were identified using the list of 26,368 RefSeq genes and gene transcripts (including 7810 immune genes) that are recorded in the GRCh37/hg19 assembly. All gene positions were binned into 40 K regions to align them with the Hi-C data. We found that the proportion of immune genes, either harboring/residing within LD block of SNPs or targeted by germline mutations remotely via inter- or intra-chromosomal interactions, was significantly higher (Fisher’s Exact Test; see Table 3 for corresponding *p*-values) for immune genes than non-immune genes. 

Note that germline mutations were not recorded for TCGA AML cohort; these observations are purely speculative and were made based on the GWAS data reported in [17]. Nevertheless, we hypothesized that germline mutations reported in [17] as reaching genome-wide significance level are highly likely to be present in TCGA AML cohort. Therefore, all genes either mutated or targeted by these germline mutations were considered as affected by germline mutations. The number of immune genes harboring somatic and germline mutations or that could be affected by germline mutations via chromatin interactions are summarized in Table 4. 

A small number of genes in each cluster (apart from Cluster 4) could potentially harbor both germline and somatic mutations. The list of these genes is given in Table 4.

### 3.6. Identification of Hub Genes

PPI data from the STRING database were used to create PPI networks for the four identified SIGs. The selected gene set for Cluster 5 was too big (2781 genes) for the STRING analysis. Around 96% of immune genes in each SIGs were involved in PPI, with the average number of interactions per gene varying between 22 and 36 (Table 5). Genes with the top 1% of interactions listed in Table 5 were considered to be important hubs in the corresponding PPI networks. Four genes—*ACTB*, *AKT1*, *TP53* and *VEGFA*—appeared to be hubs of all four networks. Unique cluster-specific hub genes were also identified (see Table 5).

## 4. Discussion

In this study we used the similarity network fusion approach [8] to integrate multi-omics profiles of 112 AML patients, comprising 24,889 DNA methylation loci, mRNA expression of 7810 immune genes and expression of 415 miRNAs, into a fused similarity network, which was subsequently subjected to spectral clustering. The optimal number of clusters was found to be five. Patients’ characteristics by cluster are given in Table 1. Analysis of the resulting clustering showed no strong correlation with either FAB or ELN subtypes/risk categories, apart from Cluster 4 in which all 11 patients were characterized as FAB M3 subtype, with ten patients having a favorable prognosis according to ELN. It is likely that patients in this cluster tend to have a favorable prognosis due to the success of all-trans retinoic acid (ARTA) treatment at targeting the *PML*::*RARA* gene fusion [24], which is distinctly present (positive) in three patients that have been tested for this gene fusion in this cluster. It was noted that patients with poor and favorable prognosis according to the ELN risk categories rarely appear in the same cluster. Note that it was impossible to compare the results with the recently proposed revised ELN classification [25]; these scores were not available for the cohort of patients analyzed. Subtyping of AML patients according to the WHO classification [3] by using differentiation markers was also not possible, since for the majority of patients these data were not available. 

Despite only modest agreement between cluster assignment and the ELN risk category, the survival analysis shows a significant (*p* = 0.00082) difference between the survival profiles of patients in different clusters (Figure 2). Although the correlation between the rankings of survival probabilities and proportions of patients in each cluster having a favorable prognosis was noted (Spearman correlation = 0.82), the *p*-value = 0.08859 did not reach the 5% level of significance. Nevertheless, one may speculate that patients’ survival/risk category is largely dependent on the similarities captured by clustering of the fused similarity network that integrates DNA methylation, mRNA expression of immune genes and expression of miRNA. Therefore, the resulting fused similarity network could be used in prediction of survival/risk categories for new patients, using, e.g., the procedure outlined in [8]. 

We suggested a way of reducing the number of features (immune genes, DNA methylation loci and miRNAs) used in constructing the fused similarity network and subsequent spectral clustering. For every cluster, we ranked each of 33,114 features with respect to their NDE index, reflecting the potential of a corresponding feature to separate patients of a given cluster from other clusters. It appeared that the use of the top 125 features from each cluster is sufficient to obtain a fused similarity network and clustering similar (with 95% accuracy) to the one obtained using all omics data available. We speculated that these top 125 features from each cluster make the most contribution to cluster separation and could be considered as the most important and informative feature in patient clustering and survival prediction. With a small loss of accuracy, this smaller fused similarity network could be used for survival/risk category prediction as outlined in [8]. 

A small number of immune genes (between three and ten) were found among top-ranked features. They included the *ALDH1A1* gene (found in Cluster 1) that emerges as a significant risk factor in AML [26]. The *HNMT* gene (Cluster 2) was found among six amino acid metabolism-related genes that correlate with the immune microenvironment and could be predictors of the prognosis and immunotherapy response of AML patients [27]. The *MPO* gene (Cluster 3) was one of the five immune genes in a model for predicting non-M3 AML patients’ treatment outcome. Note that none of the patients in Cluster 3 are classified as FAB M3 group (see Table 1). The full list of immune genes found among top-ranked features is given in Supplementary Table S3 and their role in AML from a biological point of view could be further explored. 

Epigenetic control of gene expression plays a pivotal role in determining the biological behavior of cells. DNA methylation is one such epigenetic mechanism. Not surprisingly, between 92% and 98% of features in the top 125 for each cluster were DNA methylation loci. It is known that chromatin interactions could move distal regulatory elements, promoters or the transcription start sites to the proximity required for transcription regulation of certain genes. To identify these proximal and distal targets of methylation, the set of target genes listed in TCGA database was combined with immune genes found in regions that have strong intra- and inter-chromosomal interactions with regions harboring methylation loci. 

MiRNAs are known to regulate most cellular processes and are considered promising therapeutic targets for cancer and other diseases. A small number of miRNAs (between six and 15) was found in the top-ranked features. Their potential target genes were identified using miRNet2.0 software [12] for four clusters and added to the compiled lists of genes. 

The resulting sets of genes (SIGs) were enriched in several GO biological processes/pathways terms (see Supplementary Table S4). Comparison with curated sets containing genes that up-regulated in response to α interferon proteins (M5911), γ interferon proteins (M5913) and defining inflammatory response in AML (M5932) shows that there are overlaps with selected immune genes. Further investigation and biological interpretation of these common genes may guide the finding of features responsible for survival differences between patients in the five clusters obtained. 

Further, we created PPI networks for four SIGs using the STRING database. Node degree distributions were used to identify the top 1% genes with the highest number of interactions with other proteins; these hub genes were considered to be important for a given network. Their dysregulation may influence the expression of proteins interacting with these hub genes. Four genes—*ACTB*, *AKT1*, *TP53* and *VEGFA*—appeared to be hubs of all four networks. It is known that these genes play crucial roles in the development and progression of AML, and dysregulation of these genes contributes to the proliferation, survival and chemoresistance of leukemic cells. Their aberrant expression or mutations were found to serve as important prognostic markers, influencing clinical outcomes in AML patients (see, e.g., [26,27,28,29,30]). Interestingly, all these genes were found to be targeted by various miRNAs occurring among top-ranked features, and, in some cases, by the same miRNAs within the same cluster. For example, in Cluster 1 the *ACTB* and *AKT1* genes are targets of the *hsa-mir-192*, whereas the *TP53* and *VEGFA* genes are potential targets of the *hsa-mir-106a*. Moreover, all six somatic mutations recorded in the *TP53* gene occur in patients assigned to Cluster 1. In Cluster 4, for example, three genes—*ACTB*, *AKT1* and *VEGFA*—could be targeted by *hsa-mir-1-3p*. In addition, in all five clusters the *AKT1* gene could be dysregulated by remotely acting germline mutations (possibly occurring within an enhancer) identified by GWA studies in an unrelated cohort of patients. Further understanding of “one-to-many” or “many-to-many” relationship between miRNAs and their targets is challenging but can guide the development of targeted therapies and personalized treatment approaches for AML. 

Several cluster-specific hub genes identified, such as *CASP3*, *CTNNB1*, *HIF1A*, *IL1B*, *JUN*, *MAPK1*, *UBA52* and *UBC*, are known to be involved in leukemogenesis. Dysregulation of these genes affects critical cellular processes including apoptosis, cell adhesion, transcriptional regulation, angiogenesis, inflammation and intracellular signaling. 

We identified a small number of genes in each cluster (apart from Cluster 4, in which only one patient harboring a somatic mutation was found) that could potentially mutate both in soma and germline (see Table 4). The latter mutations were identified in an unrelated cohort of patients with AML [17] via GWA studies as reaching genome-wide significance level (*p* < 5 × 10^−6^). Among these genes are the three oncogenes *KRAS*, *NRAS* (Cluster 2) and *GLI3* (Cluster 3). In turn, the *RUNX1* gene (Clusters 1 and 2) could play a dual role, being both an oncogene and tumor suppressor [31] and is considered to be an important player in AML. The platelet endothelial aggregation receptor 1 (*PEAR1*) gene (Clusters 1 and 2) could be another candidate for a tumor suppressor gene in AML [32]. One may speculate that inactivation of both alleles in these tumor suppressor genes leads to the change of a cell’s phenotype in agreement with the “two hit hypothesis” [33]. Further inspection of the other genes from this list is required to establish their tumor suppressor potential.

## 5. Conclusions

In this paper, we proposed a computational framework for finding informative and biologically plausible features that may contribute to patient similarity as identified by various clustering procedures used for patient stratification. In this study, we used spectral clustering of fused similarity networks. The advantage of using similarity network fusion is its robustness to noise, because it up-weights strong connections and down-weights weak connections in the patients’ neighborhood similarity network.

The use of this approach was demonstrated on data available for patients with acute myeloid leukemia. Further, we suggested a way of reducing the number of features used in clustering. Chromosome-conformation capture data, somatic/germline mutations and knowledge of protein–protein interactions were used to understand the observed clustering. This computational framework has the potential to guide researchers in finding plausible explanations for the features found as informative in patient’ stratification with respect to any meaningful clustering. Further experimental/biological validation is required in order to establish the functional significance of the genes found.

## Figures and Tables

**Table 1:** Patients’ characteristics by cluster.

Characteristics	Category			Cluster		
		1	2	3	4	5
**Number of patients**		27	25	29	11	20
**Gender**	male	14	13	15	5	11
	female	13	12	14	6	9
**Age**	mean	63	57	46	45	48
	median	68	58	48	42	50
	range	32–88	21–76	22–76	29–68	21–75
**Vital status**	dead	21	15	12	1	11
	alive	6	10	17	10	9
**FAB** **classification**	M0	7	0	1	0	3
	M1	8	4	9	0	10
	M2	8	0	13	0	6
	M3	0	0	0	11	0
	M4	3	10	6	0	1
	M5	0	11	0	0	0
	unclassified	1	0	0	0	0
**ELN risk** **category**	poor	15	3	5	0	6
	normal	12	20	12	1	13
	favorable	0	2	12	10	0
	not known	0	0	0	0	1
**Mutations**						
***IDH1*_r132**	positive	2	1	1	0	8
	negative	25	23	28	10	12
	not known	1	0	0	1	0
***IDH1*_r140**	positive	2	2	3	0	1
	negative	24	23	26	11	18
	not known	1	0	0	0	1
***IDH1*_r172**	positive	2	0	0	0	0
	negative	24	25	29	11	19
	not known	1	0	0	0	1
**Activating_ras**	positive	2	2	1	0	1
	negative	24	23	28	11	19
	not known	1	0	0	0	0
***NPM1c***	positive	1	14	0	0	9
	negative	25	11	29	11	11
	not known	1	0	0	0	0
***BCR::ABL1***	positive	0	0	0	0	1
	negative	1	5	2	0	2
	not known	26	20	27	11	17
***PML::RARA***	positive	0	0	0	3	1
	negative	1	2	0	0	2
	not known	26	23	29	8	17
***FLT3-ITD***	positive	1	9	7	5	10
	negative	23	16	22	6	9
	not known	3	0	0	0	1
**Total number of somatic mutations/mutated genes**		339/113	519/206	689/248	33/9	181/61

**Table 2:** The number of immune genes shared between SIGs for each cluster and gene lists corresponding to GO terms and selected curated datasets.

Number of Genes	Cluster					Total Unique
	1	2	3	4	5	
selected in cluster (SIG)	1542	1927	1328	1720	2781	4692
enriched in cytokine signaling in immune system	147	185	136	177	291	449
enriched in interferon γ response (M5913; 176 genes)	41	55	45	42	84	130
enriched in interferon α response (M5911; 97 genes)	13	27	21	13	38	56
enriched in inflammatory response (M5932; 170 genes)	53	56	42	36	78	128

**Table 3:** Number of genes harboring or targeted by remotely acting germline mutations.

Type of Interactions	Type of Genes Used	Number of Genes in Interacting Regions	Number of Genes outside Interacting Regions	*p*-Value
intra-chromosomal	immune	373	7437	8.97 × 10^−4^
	non-immune	719	17,839	
inter-chromosomal	immune	580	7230	2.19 × 10^−6^
	non-immune	1088	17,470	

**Table 4:** Immune genes in SIGs that harbor somatic and germline mutations or could be affected by germline mutations via chromatin interactions.

Number of Immune Genes	Cluster					Total Unique
	1	2	3	4	5	
in SIG	1542	1927	1328	1720	2781	4692
harboring germline mutations identified in [16]	56	86	61	79	134	214
affected by germline mutations via chromatin interactions	200	252	205	275	344	583
harboring somatic mutations	26	55	37	1	23	138
harboring both germline and somatic mutation	6	9	5	0	4	22
affected by both germline and somatic mutation	1	6	5	0	1	13
**Genes harboring or affected by both germline and somatic mutations**	**Gene symbols**					
	*AKR7A2*, *CNOT6L*, *CRISPLD2*, *GALNT2*, *PEAR1*, *RUNX1*, ***IKZF3*** ^**1**^	*CELSR3*, *KRAS*, *LARS*, *NRAS*, *PKHD1*, *PLXNA2*, *PRKCZ*, *PEAR1*, *RUNX1*, ***AP1M1***, ***CAT***, ***DLG5***, ***PLEC***, ***SMC5***, ***TPI1***	*CBL*, *GLI3*, *KLHL9*, *MYLK*, *NRXN3*, ***ADAM19***, ***FASTKD5***, ***IGF2R***, ***PCNT***, ***TRIB1***		*FLT3*, *PRDM16*, *RERE*, *XKR4*, ***CDH23***	

^1^ genes that could be affected by both germline and somatic mutation are shown in bold.

**Table 5:** Immune genes in SIGs involved in protein–protein interactions. (Note that the SIG of Cluster 5 was too big for analysis by STRING.)

Number of	Cluster			
	1	2	3	4
genes in SIG	1542	1927	1328	1720
genes in PPI (% of SIG)	1483 (96%)	1866 (97%)	1271 (96%)	1670 (97%)
connections	38,768	66,333	28,387	56,629
average connections per gene	26	36	22	34
genes with top 5% of interactions	75	94	67	84
genes with top 1% of interactions	15	20	14	17
**Genes with top 1% of interactions**	**Gene symbols**			
	*ACTB*, *AKT1*, *CCND1*, *ESR1*, ***HIF1A*** ^1^, *HRAS*, ***IL1B1***, *IL6*, *NOTCH1*, *PTEN*, *RPS27A*, *STAT3*, *TP53*, ***UBC***, *VEGFA*	*ACTB*, *AKT1*, *CD4*, ***CTNNB1***, *EGFR*, *EP300*, *ESR1*, *HRAS*, *HSP90AA1*, *HSPA8*, *IL6*, ***JUN***, *MYC*, *PTEN*, *RPS27A*, *STAT3*, *TNF*, *TP53*, ***UBA52***, *VEGFA*	*ACTB*, *AKT1*, ***CASP3***, *CCND1*, *CD4*, *EGFR*, *EP300*, *FN1*, *HSP90AA1*, *KRAS*, *MYC*, *NOTCH1*, *TP53*, *VEGFA*	*ACTB*, *AKT1*, ***CASP3***, *CCND1*, *CD4*, *EGFR*, *EP300*, *FN1*, *HSP90AA1*, *KRAS*, *MYC*, *NOTCH1*, *TP53*, *VEGFA*

^1^ genes unique to the corresponding cluster are shown in bold.

## Data Availability

Data supporting reported results can be found in Supplementary Materials available for this paper.

## Supplementary Materials

The following supporting information can be downloaded at: https://www.mdpi.com/article/10.3390/genes14091795/s1, Figure S1: Heatmap of patients’ clustering results using; Table S1: Patients’ characteristics; Table S2: Immunophenotypic characteristics of patients by cluster; Table S3: Full list of immune genes identified; Table S4: Enrichment of SIGs in GO biological processes and pathways terms.
