# Supplementary material for: Towards In Silico Identification of Genes Contributing to Similarity of Patients’ Multi-Omics Profiles: A Case Study of Acute Myeloid Leukemia
Source: Genes (Basel). 2023 Sep 13;14(9):1795. doi: 10.3390/genes14091795 (PMC10531350; doi:10.3390/genes14091795)
Supplement: Supplementary file 1 [file genes-14-01795-s001.zip › Figure S1.pdf]

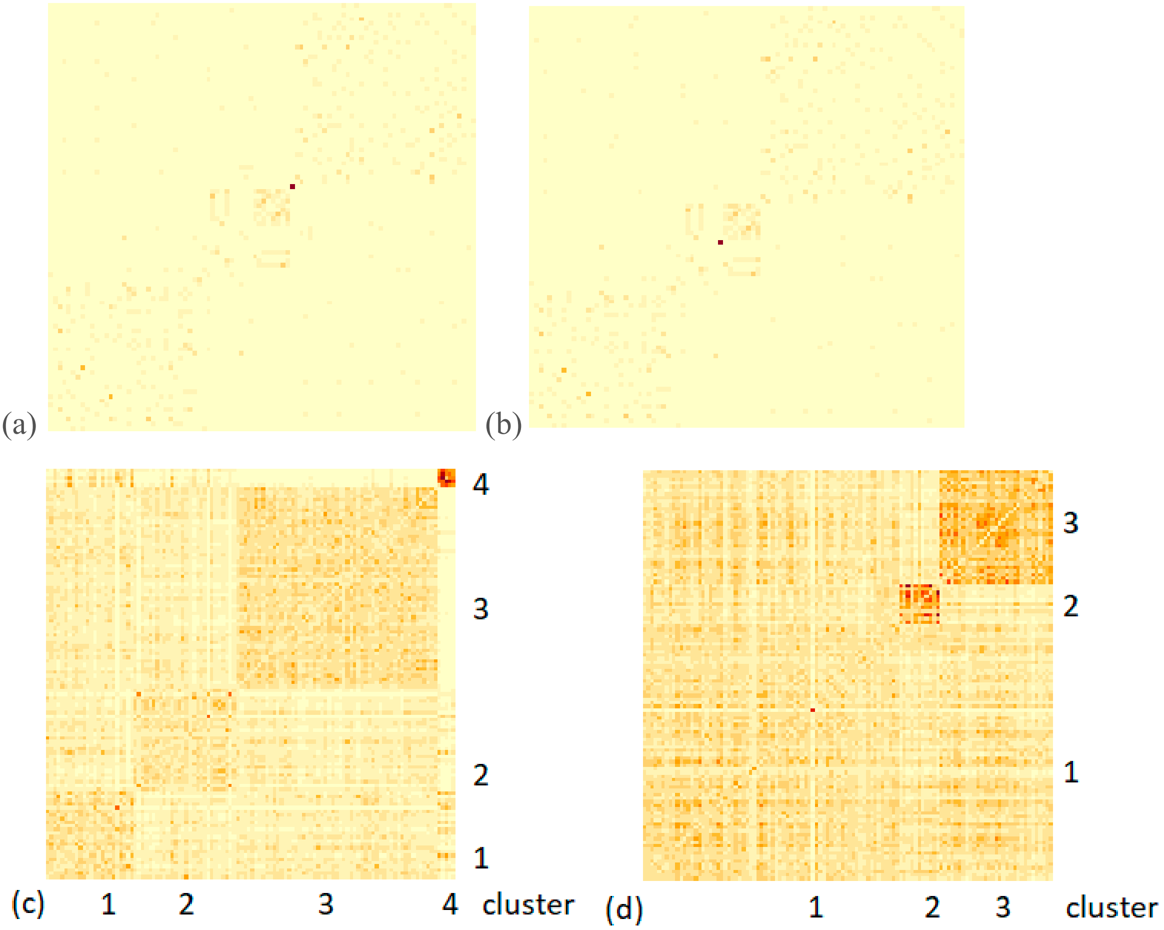

Figure S1. Heatmap of patients' clustering results using: (a) mRNA data (4 clusters); (b) mRNA data (3 clusters); (c) DNA methylation data (4 clusters) and (d) miRNA data (3 clusters). Darker colors indicate higher similarity.
