# Supplementary material for: Towards In Silico Identification of Genes Contributing to Similarity of Patients’ Multi-Omics Profiles: A Case Study of Acute Myeloid Leukemia
Source: Genes (Basel). 2023 Sep 13;14(9):1795. doi: 10.3390/genes14091795 (PMC10531350; doi:10.3390/genes14091795)
Supplement: Supplementary file 1 [file genes-14-01795-s001.zip › Table S1.pdf]

Table S1. Patients' characteristics

**Gender**	**Total**	**Mutations**	
Male	58	***IDH1*_r140**	
Female	54	positive	8
**Age**		negative	102
<60 years	68	not known	2
≥60 years	44	***IDH1*_r172**	
Median age (range)	55 (21–88)	positive	2
Average age	53	negative	108
**Vital status**		not known	2
Dead	60	**activating_ras**	
Alive	52	positive	6
**FAB classification**		negative	105
M0	11	not known	1
M1	31	***NPM1c***	
M2	27	positive	24
M3	11	negative	87
M4	20	not known	1
M5	11	***BCR*::*ABL1***	
not classified	1	positive	1
**ELN risk category**		negative	10
poor	29	not known	101
intermediate/normal	58	***PML*::*RARA***	
favourable	24	positive	4
not known	1	negative	5
**Mutations**		not known	103
***IDH1*_r132**		***FLT3*-ITD**	
positive	12	positive	32
negative	98	negative	76
not known	2	not known	4
