# Supplementary material for: Towards In Silico Identification of Genes Contributing to Similarity of Patients’ Multi-Omics Profiles: A Case Study of Acute Myeloid Leukemia
Source: Genes (Basel). 2023 Sep 13;14(9):1795. doi: 10.3390/genes14091795 (PMC10531350; doi:10.3390/genes14091795)
Supplement: Supplementary file 1 [file genes-14-01795-s001.zip › Table S2.pdf]

Table S2. Immunophenotypic characteristics of patients by cluster.

Characteristic	Test result	Cluster				
		1	2	3	4	5
CD3	not tested	25	25	28	11	20
	positive	0	0	0	0	0
	negative	2	0	1	0	0
CD5	not tested	21	19	22	9	15
	positive	1	0	1	0	0
	negative	5	6	6	2	5
CD7	not tested	25	25	28	11	17
	positive	2	0	1	0	1
	negative	0	0	0	0	2
CD10	not tested	27	25	28	11	20
	positive	0	0	0	0	0
	negative	0	0	1	0	0
CD14	not tested	21	11	20	7	12
	positive	0	4	1	0	0
	negative	6	10	8	4	8
CD15	not tested	27	25	29	11	19
	positive	0	0	0	0	0
	negative	0	0	0	0	1
CD19	not tested	21	21	20	8	14
	positive	0	0	2	0	0
	negative	6	4	7	3	6
CD20	not tested	26	25	29	11	20
	positive	0	0	0	0	0
	negative	1	0	0	0	0
CD23	not tested	27	24	29	11	20
	positive	0	1	0	0	0
	negative	0	0	0	0	0
CD25	not tested	26	25	29	11	20
	positive	0	0	0	0	0
	negative	1	0	0	0	0
CD33	not tested	10	1	3	0	1
	positive	14	24	22	11	18
	negative	3	0	4	0	1
CD34	not tested	1	9	1	1	0
	positive	25	10	28	1	13
	negative	1	6	0	9	7
CD45	not tested	25	24	26	11	17
	positive	2	1	3	0	3
	negative	0	0	0	0	0
CD56	not tested	19	13	18	8	12
	positive	2	4	4	0	2
	negative	6	8	7	3	6
CD117	not tested	22	5	3	0	2

	positive	5	20	26	11	18
	negative	0	0	0	0	0
HLA-DR	not tested	13	10	11	3	6
	positive	12	15	18	0	8
	negative	2	0	0	8	6
MPX	not tested	1	2	3	1	2
	positive	18	18	25	10	17
	negative	8	5	1	0	1
NSE	not tested	3	2	5	3	1
	positive	2	19	7	1	1
	negative	22	4	17	7	18
other_CD	not tested	20	8	20	8	11
	positive	1	11	2	1	0
	negative	6	6	7	2	9
TdT	not tested	24	25	25	11	18
	positive	2	0	2	0	2
	negative	1	0	2	0	0

Abbreviations

CD	cluster of differentiation markers (for full list and explanation see https://wiki.clinicalflow.com/cluster-differentiation-cd-markers)
HLA-DR	a molecule typically expressed by antigen-presenting cells and is associated with antigen presentation. Expressed on T cells and has been associated with T cell activation.
MPX	Myeloperoxidase (MPO) is a marker of myeloid differentiation
NSE	Neuron-specific enolase is a substance that has been detected in patients with various tumors.
TdT	Terminal deoxynucleotidyl transferase is a nuclear protein widely used as a marker for the diagnosis and classification of acute leukemia
